# Long COVID Through a Public Health Lens: An Umbrella Review

**DOI:** 10.3389/phrs.2022.1604501

**Published:** 2022-03-15

**Authors:** Vasileios Nittas, Manqi Gao, Erin A. West, Tala Ballouz, Dominik Menges, Sarah Wulf Hanson, Milo Alan Puhan

**Affiliations:** ^1^ Department of Epidemiology, Epidemiology, Biostatistics and Prevention Institute, Faculty of Medicine, University of Zurich, Zürich, Switzerland; ^2^ Department of Environmental Systems Sciences, ETH Zürich, Zürich, Switzerland; ^3^ Institute for Health Metrics and Evaluation, University of Washington, Seattle, WA, United States

**Keywords:** COVID-19, post COVID-19, public healh, prevalence, review, long COVID

## Abstract

**Objectives:** To synthesize existing evidence on prevalence as well as clinical and socio-economic aspects of Long COVID.

**Methods:** An umbrella review of reviews and a targeted evidence synthesis of their primary studies, including searches in four electronic databases, reference lists of included reviews, as well as related article lists of relevant publications.

**Results:** Synthesis included 23 reviews and 102 primary studies. Prevalence estimates ranged from 7.5% to 41% in non-hospitalized adults, 2.3%–53% in mixed adult samples, 37.6% in hospitalized adults, and 2%–3.5% in primarily non-hospitalized children. Preliminary evidence suggests that female sex, age, comorbidities, the severity of acute disease, and obesity are associated with Long COVID. Almost 50% of primary studies reported some degree of Long COVID-related social and family-life impairment, long absence periods off work, adjusted workloads, and loss of employment.

**Conclusion:** Long COVID will likely have a substantial public health impact. Current evidence is still heterogeneous and incomplete. To fully understand Long COVID, well-designed prospective studies with representative samples will be essential.

## Introduction

Long COVID is a novel syndrome that is broadly defined by the persistence of physical and/or psychological and cognitive symptoms following a probable or confirmed SARS-CoV-2 infection, usually 3 months from acute infection and lasting longer than 2 months, with no probable alternative diagnosis [1, 2]. The literature provides a very diverse set of descriptions and definitions. Some of the commonly used terms include “long haulers,” “post-acute COVID-19,” “persistent COVID-19 symptoms,” “post COVID-19 manifestations,” “post COVID-19 syndrome,” “chronic COVID-19 syndrome,” “post-infectious COVID-19,” “post-acute sequelae of SARS-CoV-2 infection,” and “post COVID-19 recovery syndrome” [1, 3–7]. The World Health Organization (WHO) now uses the term Post COVID-19 condition [2]. The National Institute for Health and Care Excellence (NICE) guidelines classifies Long COVID in two categories: 1) “ongoing symptomatic COVID-19” for symptoms lasting from four to 12 weeks and 2) “Post-COVID-19 syndrome” for persisting symptoms beyond 12 weeks after disease onset; both categories only hold if symptoms cannot be explained by alternative diagnoses [1, 6, 8, 9]. The National Institute for Health Research (NIHR) emphasizes that Long COVID might not be a single condition, but multiple syndromes, such as the post-intensive care syndrome, post-viral fatigue syndrome, and long-term COVID syndrome [1]. Those affected describe impairing, debilitating, and complex symptoms, sometimes keeping them out of work and social life [10]. To fully understand Long COVID and inform crucial healthcare and policy responses, it is key to understand its public health implications.

### Aims and Research Questions

This study aimed to provide a summary of existing evidence on the public health implications of Long COVID, focusing on clinical, epidemiological, and socio-economic aspects. We addressed the following questions:

a) What are the reported Long COVID symptoms, risks, and protective factors?
b) What are the current prevalence estimates of Long COVID?
c) What are the potential social and economic implications of Long COVID?

## Methods

We used a two-stage methodology consisting of an umbrella review and a targeted evidence synthesis of the included primary studies. The first research question was answered with information reported in reviews, while the second and third with information reported in the primary studies.

### Umbrella Review

The first stage consisted of a review of reviews (umbrella review) following PRISMA guidelines [11]. We searched the following electronic databases: Medline (EBSCOhost), CINAHL (EBSCOhost), WHO COVID-19 (including Elsevier, MedRxiv), and Embase (excluding Medline). We developed a sensitive search strategy using terms related to COVID-19 and long-term consequences. The detailed strategy is provided in Supplementary File S1. Keywords were combined and refined using Boolean operators and truncations, adjusted to each of the databases. We additionally searched Google Scholar, screening the first five result pages. Finally, we manually screened the reference lists of all included reviews. All references were screened in duplicate, at title and abstract, as well as full-text level. All searches were conducted on 15 March, 2021, and updated on 9 July, 2021. The eligibility criteria for reviews are listed in Table 1.

**TABLE 1:** Eligibility criteria for reviews and primary studies (Long COVID through a public health lens: An Umbrella Review. Switzerland 2021).

Eligibility criteria for reviews
Reported a review methodology (systematic or scoping reviews, rapid reviews, pragmatic reviews)
Thematically focused (entirely or partially) on Long COVID
**Eligibility criteria for primary studies**
Included in one of the reviews or identified through a related article search
Must be surveys, cross-sectional or cohort studies including laboratory or clinically confirmed SARS-CoV-2 cases for at least 6 weeks (from acute disease, test, hospital discharge, enrollment, or study start)

### Evidence Synthesis of Primary Studies

The second stage consisted of an evidence synthesis of primary research. First, we identified all primary studies included in at least one of the eligible systematic reviews. Second, using those primary studies, we conducted related article searches in PubMed and Google Scholar, capturing newer primary studies that have not yet been included in one of our reviews. We then included and synthesized primary studies from both stages that fulfilled all eligibility criteria. Data synthesis for primary studies was focused on 1) the prevalence and 2) the socio-economic impact of Long COVID, as these two elements were not adequately addressed in systematic reviews. Searches were conducted in May 2021. The eligibility criteria for primary studies are listed in Table 1. To capture the topic’s emerging nature, we decided to also include primary studies at preprint stages. All preprint studies are marked with a hashtag and should be viewed with caution, as peer review might lead to substantial revisions. All preprint findings should be considered provisional. To assess the impact of preprints on the prevalence estimates reported, we temporarily deleted them and compared ranges and median values with and without preprints.

### Data Extraction and Synthesis

Review data was extracted with a predefined data extraction sheet including methodological characteristics (type of review, number of included primary studies, socio-demographic focus, geographic distribution of primary studies) and three different sections, each corresponding to one of the research questions. Primary study data was extracted with a separate, predefined sheet including information on study design, sample size, recruitment period, the severity of acute SARS-CoV-2 infection, sample socio-demographics, follow-up lengths, socio-economic implications, and prevalence estimates. Data extraction was conducted by one reviewer and validated by three reviewers. EAW validated all data regarding prevalence estimates. TB and DM quality-checked parts of the risk of bias assessments.

In accordance with the NICE guidelines [9], prevalence estimates for adults were only reported for studies with a mean follow-up at 12 weeks or above. For children, we report prevalence estimates at 4 weeks and beyond, as estimates at 12 weeks and beyond are currently scarce. We only provided a detailed report of prevalence estimates derived from studies with population-based samples and/or control participants, as these studies are more likely to yield more robust and less biased estimates. We report prevalence estimates according to the study’s source population (hospitalized, non-hospitalized, or mixed) and age groups (adults, children). Studies were classified as population-based if they were based on sampling procedures that are widely accepted to yield representative samples (e.g., probability sampling or census data). For studies with control groups, we report adjusted prevalence estimates (difference between the estimate for cases and estimate for controls).

### Risk of Bias (Quality) Assessment

The quality of reviews was assessed using the AMSTAR (Assessing the Methodological Quality of Systematic Reviews) checklist [12]. The quality of primary studies that report prevalence estimates (≥12-week follow-up for adults, ≥4-week follow-up for children) was evaluated with three items, adapted from the Hoy et al. [13]. The first item assessed whether the target population is a good representation of the national population. The second determined whether the sample was selected with some form of random and/or consecutive procedure. The third item assessed whether the likelihood of non-response bias was minimized.

## Results

**FIGURE 1:**
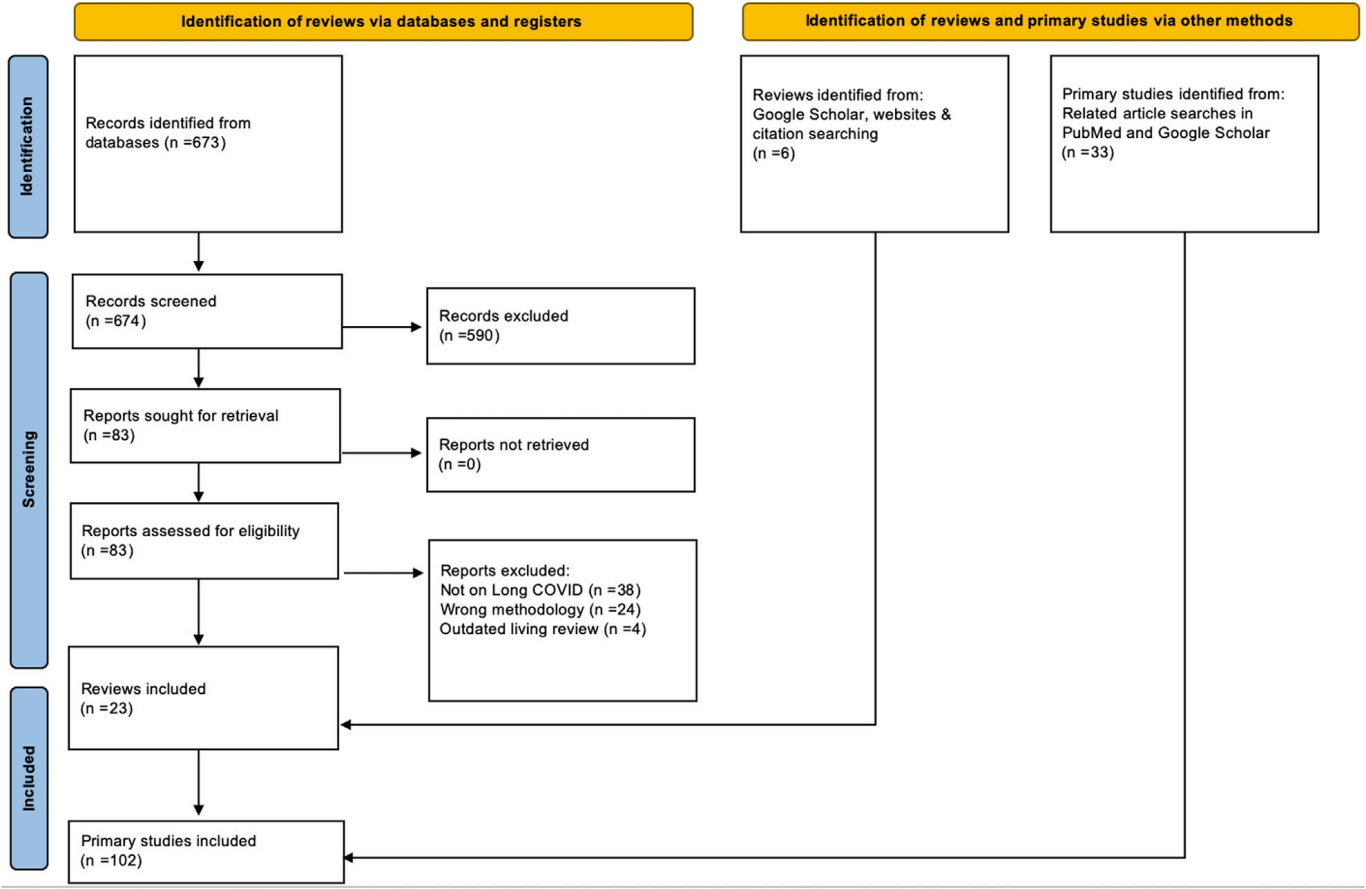
PRISMA Flowchart for reviews and primary studies (Long COVID through a public health lens: An Umbrella Review. Switzerland 2021).

Our database searches yielded 673 references. 590 of those were excluded at title and abstract screening, and 83 manuscripts were screened full-text. This led to the exclusion of 66 further references. Google Scholar and reference list searches yielded an additional five references, leading to 22 included reviews. During the second review stage, we included 102 primary studies, 69 of them retrieved from the 22 previously identified reviews and 33 identified through related article searches in PubMed and Google Scholar. Figure 1 provides the PRISMA flowchart of all our searches and screening processes.

### Characteristics of Included Studies

One review was published in 2020 and 21 in 2021. Most were traditional systematic reviews (*n* = 11), followed by rapid reviews (*n* = 2), rapid living systematic reviews (*n* = 2), pragmatic reviews (*n* = 3), systematic reviews with a meta-analyses (*n* = 3), and one scoping review. The overall quality of included reviews was assessed as low to moderate, with eight scoring critically low, four scoring low, 10 scoring moderate, and one scoring high in quality. The full quality assessment is provided in Supplementary File S2.

Most primary studies (*n* = 60) were published in 2020, followed by 42 publications in 2021. Most primary research was based on prospective cohorts (*n* = 71), followed by cross-sectional and survey designs (*n* = 19), retrospective cohorts (*n* = 10), case series, and case-control studies (*n* = 2). Most primary studies included hospital-based samples (*n* = 48), seventeen enrolled non-hospitalized participants, while the remaining 37 included mixed samples.

### Symptoms, Risks, and Protective Factors

#### Symptoms and Manifestations

**FIGURE 2:**
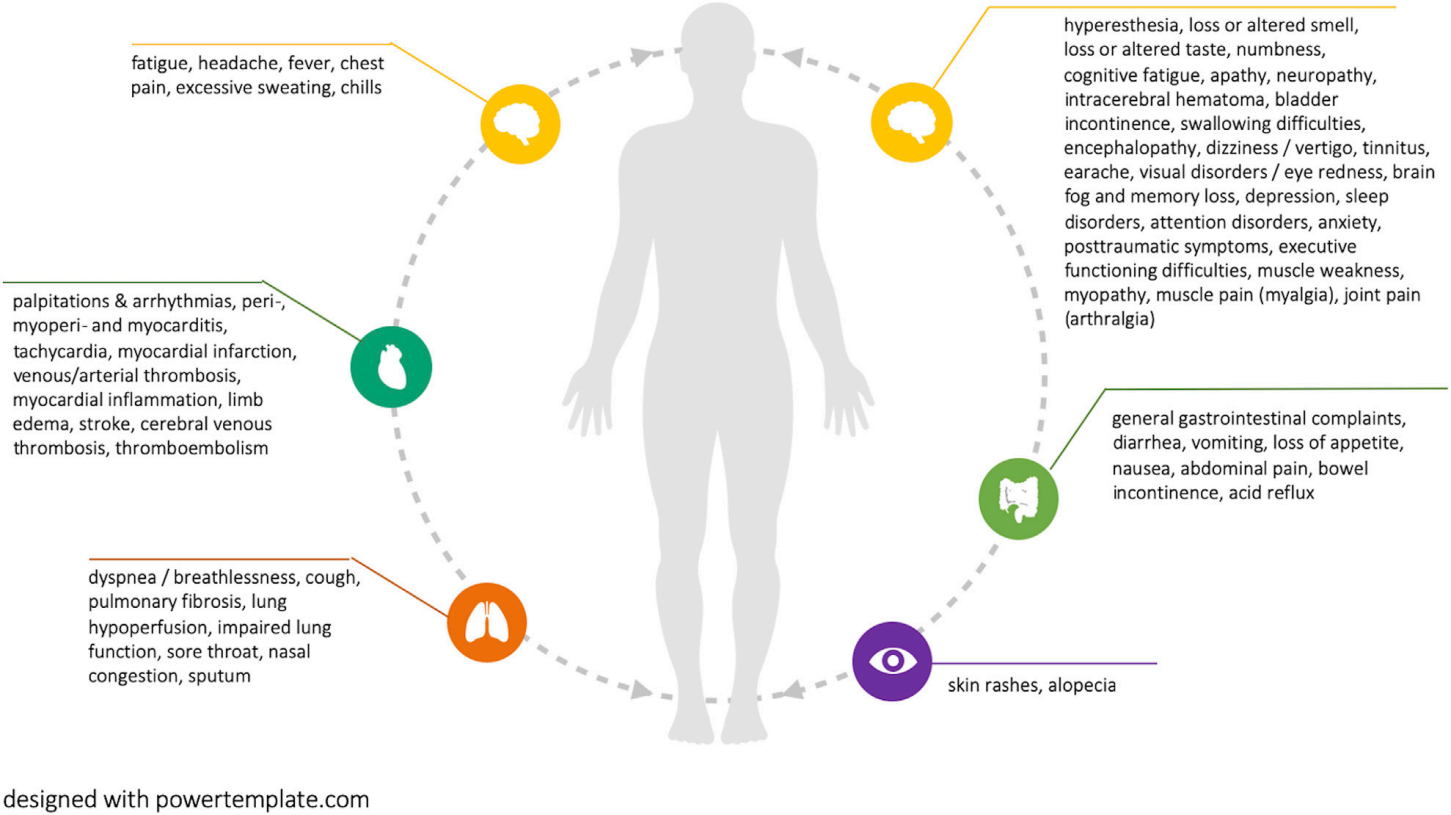
Reported potential Long COVID symptoms (Long COVID through a public health lens: An Umbrella Review. Switzerland 2021).

Current reviews report more than 50 distinct symptoms that are potentially associated with Long COVID (see Figure 2). The most prevalent symptoms were fatigue and breathing difficulties, followed by smell and taste disturbances, headache, chest pain, brain fog and memory loss, as well as sleep disorders. Figure 2 visualizes all reported potential Long COVID symptoms. Supplementary File S3 additionally provides the number of reviews in which each symptom is reported.

One review reported that Long COVID symptoms can occur in symptom clusters, while some patients experience multiple and multi-system outcomes [1]. Two reviews did highlight that Long COVID can have a relapsing-remitting nature, characterized by periods of improvements and flare-ups [1, 6]. Long COVID symptoms were often reported as debilitating, having a strong negative impact on mental health and quality of life [1, 6, 7]. Eight reviews highlighted emerging evidence of potentially associated organ impairment, primarily of the heart, lungs, kidneys, and brain, with associations remaining ambiguous. The evidence for pediatric Long COVID patients remains limited. However, there are indications of multisystem inflammatory syndrome development, as well as a range of symptoms that are also common among adults, including fatigue, breathing difficulties, heart palpitations, headaches, concentration difficulties, and cognitive deficits, muscle weakness and pain, dizziness, sore throat, abdominal pain, depression, and skin rashes [14]. Most existing reviews did not classify disease and symptom severity based on indicators such as the number of medical visits or inability to work. These are important indicators, which, if combined with lived experience of symptoms, their duration, as well as their interference with social life can provide a holistic picture of disease burden.

### Risk and Protective Factors

Some studies suggested that the following factors might increase the risk for Long COVID development: 1) female sex, 2) age, 3) comorbidities (mental and physical), 4) severity of acute disease (e.g., hospitalization, higher chest imaging scores, duration of oxygen supplementation, pneumonia), and 5) obesity [1, 3, 6, 8, 14]. For some of these factors, the evidence seems to be inconsistent. Three reviews reported that individuals experiencing more than five symptoms during acute disease—among which fatigue, headache, dyspnea, chest pain, sensitive skin, hoarse voice, and myalgia—had a higher risk of subsequently developing Long COVID [6, 7, 15, 16]. Psychological symptoms, especially those associated with posttraumatic stress, seem to be affecting younger people, women, and those with responsibilities for others [7]. Beyond physical activity level [3], none of the reviews reported on protective factors regarding the development of Long COVID.

### Prevalence of Long COVID

Current Long COVID prevalence estimates vary. This is due to large methodological variation of primary studies, including their sample recruitment methods (e.g., hospital, non-hospital, self-selection), follow-up periods, definitions of Long COVID and the distinction between symptoms directly related to SARS-CoV-2 from unrelated symptoms (e.g., from pre-existing conditions) [1]. Thus, all current estimates need to be viewed with caution and along with their respective definitions.

We identified 40 out of 102 (39%) primary studies that reported prevalence estimates of Long COVID or some of the associated symptoms. Thirteen studies included population-based samples and/or control groups and are reported in detail. Prevalence estimates reported in the 27 primary studies without control groups or population-based samples are provided in Supplementary File S4.

### Adults

We identified 10 population-based and/or control group studies reporting Long COVID prevalence estimates (≥12 week follow-up) in adults, summarized in Table 2.

**TABLE 2:** Reported prevalence estimates for adults (Long COVID through a public health lens: An Umbrella Review. Switzerland 2021).

Authors (References)	Cases (n =)	[% hospitalized]	Controls (*n* =)	Follow-up period [follow-up start]	Symptom prevalence cases (%)	Symptom prevalence controls (%)	Adjusted prevalence (% cases—% controls)
**Study sample: non-hospitalized adults**							
Stavem et al. [17] [p]	451	NA	NA	6–24 [positive test]	41	—	—
Graham et al. [18] [c]	100	NA	50	18 – 23 [symptom onset]	67.8	60.3	7.5
Havervall et al. [19] [c]	323	NA	1027	≥32 [January 2020]	15	3	12
^a^Desgranges et al. [20] [c]	418	NA	89	12–40 [acute disease]	53	37	16
**Study sample: hospitalized and non-hospitalized adults**							
Menges et al. [21] [p]	431	19	NA	29 [acute disease]	26	—	—
Petersen et al. [22] [p]	180	4	NA	18 [acute disease]	53.1	—	—
Sudre et al. [23] [c]	4182	14	4182	≥12 [symptom onset]	2.3	—	—
^a^Cirulli et al. [24] [c]	357	3	5497	12 [January 2020]	14.8	7	7.8
Logue et al. [25] [c]	177	9	21	12–36 [symptom onset]	32.8	4.8	28
**Study sample: hospitalized adults**							
Xiong et al. [26] [c]	538	100	184	>12 [hospital discharge]	49.6^b^	12	37.6

a = still at preprint stage at time of data extraction; P, population-based sample; C, includes control participants; NA, not applicable.

b Study provides multiple prevalence estimates, according to symptom groups. 49.6% is the highest reported prevalence (generally symptoms).

### Non-Hospitalized Adults

Only one study included a population-based sample of exclusively non-hospitalized participants, reporting a prevalence 41% [17]. The study included 451 previously diagnosed participants from a large catchment area of two hospitals in Norway. The sample was predominantly female and over 50 years of age, and overall findings were subject to risk of recall bias. The remaining estimates for non-hospitalized adults come from studies with control groups, however without population-based samples. Three studies included only non-hospitalized participants, reporting estimates of 7.5% [18], 12% [19], and 16% [20]. Some of their limitations include 1) small sample sizes, 2) the use of serology testing, not allowing for an accurate identification of infection start and 3) recall bias.

### Hospitalized and Non-Hospitalized Adults

Two studies included population-based samples with hospitalized, as well as non-hospitalized participants, reporting Long COVID prevalence rates of 26% [21] (19% hospitalized) and 53.1% [22] (4% hospitalized). Both studies did not assess pre-COVID physical or mental health, and thus are not able to accurately distinguish between COVID-19-related and pre-existing symptoms. Three studies included control groups and hospitalized, as well as non-hospitalized, participants reporting estimates of 2.3% [23] (14% hospitalized), 7.8% [24] (3% hospitalized) and 28% [25] (9% hospitalized). Some of their limitations include 1) not differentiating between symptoms occurring before and after test results, 2) large losses to follow-up, 3) small sample sizes and 4) recall bias. The very low estimate (2.3%) might be due to lacking representation of elderly subgroups (>70) and the interference of Long COVID symptoms while using the app [23].

### Hospitalized Adults

Finally, one study included a sample of only previously hospitalized participants, reporting an estimate of 37.6% [26]. The study enrolled 538 previously hospitalized SARS-CoV-2 cases from a single hospital in Wuhan (discharged by March 1st, 2020) as well as 184 healthy non-hospitalized controls [26]. The study included primarily severe cases and was subject to risk of recall bias.

### Children and Teenagers

We identified three population-based and/or control group studies reporting Long COVID prevalence estimates (≥4-week follow-up) in children and teenagers, summarized in Table 3.

**TABLE 3:** Reported prevalence estimates for children and teenagers (Long COVID through a public health lens: An Umbrella Review. Switzerland 2021).

Authors (References)	Cases (n =)	[% symptomatic; % hospitalized]	Controls (n =)	Follow-up period [follow-up start]	Symptom prevalence cases (%)	Symptom prevalence controls (%)	Adjusted prevalence (% cases—% controls)
**Non-hospitalized children**							
Radtke et al. [27] [p; c]	109	NA	1246	>12 [October 2020]	4	2	2
^a^Miller et al. [28] [c]	175	NA	4503	≥4 [February 2020]	4.6	1.7	2.9
**Hospitalized and non-hospitalized children**							
Molteni et al. [29] [c]	1734	2	1734	≥4 [symptom onset]	4.4	0.9	3.5

a = still at preprint stage at time of data extraction; P = population-based sample; C = includes control participants; NA , not applicable.

### Non-Hospitalized Children

One study explored Long COVID in non-hospitalized children, reporting prevalence estimates of 2% and 2.9%. The first was the Swiss Ciao Corona cohort, a population-based study that explored the long-term symptoms (>12 weeks) after a SARS-CoV-2 infection in school children. The sample of 109 seropositive children and 1246 seronegative controls was recruited through a randomly selected sample of 55 schools across the canton of Zurich [27]. Based on seroprevalence, the study does not distinguish between symptoms before and after SARS-CoV-2 infection, as the actual time points of infection were not assessed. The study’s small sample size is an additional limitation. The second study was a household cohort study in England and Wales, including 173 children with a history of SARS-CoV-2 infection and 4503 controls. Again, the small number of cases is a strong study limitation [28].

### Non-Hospitalized and Hospitalized Children

Two studies explored Long COVID in a sample of non-hospitalized, as well as hospitalized (3%) children, reporting a prevalence of 3.5% [29]. The study was based on UK data of children aged five to 17 years, retrieved from the COVID Symptom study. The sample consisted of 1734 cases and an equal number of controls, either reported by adult contributors (by proxy) or teenagers aged 16–17 years. The study’s design (symptom questions) was primarily informed by research in adults, while the mobile self-reporting nature might have introduced self-report bias and other errors.

### Risk of Bias Assessment for Studies Reporting Prevalence Estimates

Regarding our risk of bias assessment, only three studies scored “low risk” for the first item (“is the target population representative of the national population”), three studies scored “low risk” for the second item (“is some sort of random selection used to select the sample”), and five scored “low risk” for the third item (“is the likelihood of non-response bias minimized”) [13]. Supplementary File S5 provides a summary of all risk of bias scores for studies with control groups and/or population-based samples (for all studies listed in Tables 1, 2). Deleting all preprints did not have any substantial impact on our findings (prevalence estimates or median values).

### Social and Economic Implications

#### Family Life and Social Functioning

About 29% (*n* = 29) of all included primary studies reported some degree of daily life, family, and social functioning, as well as quality of life impairment related to Long COVID [18, 19, 21, 25, 26, 30–53]. Many reported functional restrictions that often require lifestyle changes, changes in physical activity levels, restricted social life, and role limitations [34, 37, 38, 54]. Neurological, cognitive, and psychological symptoms, such as anxiety or memory impairment, strongly impact daily living and quality of life, while routine activities, such as driving and cooking can become very difficult or even impossible [25, 26, 35, 39]. Two cohort studies reported that 12% (*n* = 1250) and 44% (*n* = 100) of their participants had difficulties or were unable to perform usual daily activities at about 2 months after being hospitalized with a SARS-CoV-2 infection [50, 55]. This is also the case for those living with Long COVID after mild to moderately severe infections, with studies reporting that about 50% of their participants were facing daily activity impairments after 2 months and 5 months [39, 40], and about 15% still reporting social and home disruptions 8 months after disease onset [19, 50, 55].

For some, even those who were completely independent before, these limitations are often severe enough that they required daily assistance, or had at least some form of dependency [19, 49–51]. At 8 months after mild acute infection, 11% of 323 Swedish cohort participants reported some degree of disruption in at least one disability scale category [19]. Two cohort studies, both following up previously hospitalized participants for about 2 months, reported that 16% of participants faced reduced self-care capacity due to Long COVID [50, 52]. A US-based case series with a sample size of 247 previously hospitalized SARS-CoV-2 patients reported that about one-third of participants required post-acute care and indicated some form of dependency [49]. A cross-sectional observational study of 183 previously hospitalized patients (6-month follow-up) in Spain reported significant everyday life functioning limitations among 56% of intensive care unit patients and 17.9% among individuals not requiring intensive care [45].

### Work-Related Implications

Inevitably, Long COVID is also expected to have a considerable impact on the workforce. About 13% (*n* = 13) of all included primary studies reported employment-related consequences of Long COVID [19, 26, 35, 39, 40, 44, 47, 50, 55–59]. In studies on previously hospitalized participants, absence from work due to Long COVID was reported in 9%–40% of those previously employed at two to 3 months after discharge [50, 55, 56, 59]. Research on primarily mild to moderate and non-hospitalized SARS-CoV-2 cases reported that about 12%–23% remained absent from work (or had long absence periods) at three to 7 months after acute disease [35, 39]. A cohort study with a mixed sample (hospitalized and non-hospitalized) reported that 70% of participants were absent from work for a period of 13 weeks or more, while another one (hospitalized and non-hospitalized) reported that 31% were still out of work at 6 weeks after acute illness [40, 57]. Besides full absence, studies reported that many of those living with Long COVID are forced to adjust or reduce their workload. Two cohort studies following up on previously hospitalized participants for about 2 months reported that 15% and 40% of their employed participants adjusted their employment to their current circumstances [50, 55]. At follow-up of three to 8 months, proportions ranged from 8% to 45% for previously mild to moderate cases [19, 35, 39]. Finally, two studies reported permanent employment loss in relation to deteriorating health, with one reporting that 11% and the other 13.8% of their previously employed participants were unemployed at 2 months after acute disease [55, 58].

## Discussion

Given the recent emergence of Long COVID and the premature state of ongoing research, the current literature inevitably provides a still patchy, heterogeneous, and thus inconclusive picture of its overall burden and broader public health implications. This heterogeneity is well reflected in currently reported prevalence estimates for adults, which range from 7.5% to 41% for samples of non-hospitalized participants, and 2.3%–53% for samples with hospitalized and non-hospitalized participants. This is due to several factors and currently prevailing methodological limitations.

First, much of the earlier research on SARS-CoV-2 was designed and implemented quickly at the onset of the pandemic, with a focus on conveniently sampled hospital and outpatient participants, rather than larger, randomly sampled studies. The samples recruited during the early phase of the pandemic were also often not as widespread and captured cases were likely more severe. We only identified four population-based studies reporting prevalence estimates.

Second, the prevalence of certain symptoms is rarely placed in relation to their prevalence in persons without SARS-CoV-2 infection before or during the pandemic. Most studies fail to accurately distinguish between SARS-CoV-2 related symptoms to those linked to other (often pre-existing) conditions. This is particularly important for severe and potentially life-threatening outcomes that involve vital organ impairment, such as the heart or lungs. Most primary studies reporting organ involvement are not able to unambiguous associations yet [34, 40, 56]. Third, certain population subgroups, including the elderly, people with disabilities, children as well as a large proportion of asymptomatic SARS-COV-2 patients remain underrepresented [1]. Only three of all primary studies in this review provided some estimates on the prevalence of Long COVID in children and teenagers, ranging from 2% to 3.5%. These estimates need to be viewed with caution as they are largely derived from very small samples and bias-prone reporting methods.

While evidence on risk factors is still emerging, only one review discussed the protective factors, suggesting that good physical activity might reduce the risk of developing Long COVID [3]. Research on protective factors and risk-mitigating behaviors, as well as on the mechanisms through which different treatment options may potentially affect the risk for and severity of Long COVID will be key to future prevention approaches. Similarly, as vaccines become widespread globally, research will need to shift towards understanding the effects (if any) of vaccination on Long COVID symptoms, and whether any long-term protective effect exists for those who are vaccinated and still contract SARS-CoV-2.

The identified evidence demonstrates that Long COVID can have debilitating consequences on quality of life, social and family life, as well as on employment. Many of those living with Long COVID face various degrees of impairment and disability, impacting daily living, social functioning, and mental health. Similarly, many affected individuals face longer periods off work, reduced working hours, and potentially higher risk of unemployment and financial hardship, adding to an overall socio-economic burden. While there is no clear evidence regarding the broader economic implications of Long COVID, there is evidence that it affects a significant proportion of the formerly healthy working population, which may lead to long-term economic consequences as well as healthcare system strains [1, 54]. The long-term economic burden of a substantially large affected population will emerge over time and is expected to have a heavy impact on healthcare utilization costs.

Overcoming remaining uncertainties will ultimately require some future studies with some of the following methodological features: 1) large, prospective population-based samples and representative control groups, 2) carefully captured symptoms before infection, 3) stronger emphasis on potentially protective factors, 4) inclusion of socio-economic data, 5) and longer follow-up times (>12 months). These should be complemented by qualitative studies that capture the lived experiences of people with Long COVID. Fully understanding such complex and multifaceted health conditions requires approaches that capture and amplify the voices of those affected. Citizen science projects, co-designed with those affected provide an ideal medium to capture that additional, yet much-needed perspective.

Based on the findings of this review, we believe that the public health implications of Long COVID are equally multi-faceted as Long COVID itself. Its complex manifestation, but also its broad range of severity, requires interdisciplinary and holistic healthcare approaches. The National Health Service (NHS) of the UK may pave the way for other countries by providing information, support, and care at different levels for patients and providers. For example, the website https://yourcovidrecovery.nhs.ch informs about the condition and its many manifestations, and provides easily accessible practical support for patients and families. The NHS together with NICE early on guided the diagnosis and management of Long Covid in primary care. Primary practitioners will likely be able to care for most patients with Long Covid and act as gatekeepers, which is particularly important for a multifaceted condition like Long Covid. Finally, those with complex clinical manifestations can be referred to an NHS Long Covid clinic, where an interdisciplinary team provides thorough assessments and disease management plans. Such a concerted effort does require significant resources but appears promising to counter the under- and overtreatment of Long Covid. The challenges around Long Covid go beyond medical needs. Although not yet clear, preliminary evidence suggests the socio-economic implications of Long COVID can be substantial. For those most affected, the financial constraints that come with lost or reduced employment may further negatively impact overall well-being and mental health. That will ultimately require broader safety nets and support structures in place, ensuring that those affected by Long COVID are not discriminated against or further disadvantaged.

The findings of this review need to be viewed with the following limitations in mind. The heterogeneity and premature state of current research does not allow for confident statements on Long COVID’s prevalence, neither on its broader public health nor an economic burden. We navigated through this uncertainty in a narrative and structured way, highlighting those studies that provided the highest methodological robustness. While we developed a sensitive research strategy and believe to have captured all reviews published at that point in time, this is a rapidly emerging topic, for which our findings merely provide a snapshot of preliminary evidence. Primary studies were extracted from included systematic reviews and not captured through a separate search strategy, which inevitably excludes those that have been published after the systematic reviews. We counteracted that limitation through additional related article searches in PubMed and Google Scholar, ensuring an updated and representative sample of available primary evidence. In line with NICE’s classification, we only included primary studies with follow-up periods at 6 weeks or beyond and only reported prevalence estimates at 12 weeks and beyond. In light of a lacking commonly agreed-upon definition of Long COVID, prevalence estimates should always be interpreted in relation to the chosen follow-up periods and definitions used in the respective studies. A prospective protocol of this review has not been registered or published.

### Conclusion

Our review summarizes the current evidence on the prevalence of Long COVID among previously infected individuals and outlines the multifaceted nature of its symptoms, as well as the remaining uncertainty around their progression, underlying risk factors, and broader socio-economic implications. As primary studies and reviews emerge at a fast pace, current evidence is inevitably bound by methodological variation and limitations. Improving our knowledge of Long COVID will ultimately require well-designed prospective studies, with clearly reported Long COVID definitions, accurate distinction of SARS-CoV-2-related symptoms, and adequate follow-up times. Representative and large samples, across severity levels of acute infection, are vital along with the inclusivity of currently underrepresented groups, including children and various minorities. This shall be accompanied by qualitative, person-centered research, ensuring that the full complexity of living with Long COVID is explored and understood.
