# Supplementary material for: Long COVID Through a Public Health Lens: An Umbrella Review
Source: Public Health Rev. 2022 Mar 15;43:1604501. doi: 10.3389/phrs.2022.1604501 (PMC8963488; doi:10.3389/phrs.2022.1604501)
Supplement: Supplementary file 1 [file DataSheet3.docx]

| Supplementary file 3. Reported Long COVID Symptoms and number of reviews each symptom has been reported in (Long COVID through a public health lens: An Umbrella Review. Switzerland 2021)     \| **Symptoms (number of reviews reporting symptom)** \| \| --- \| \|  \| \| **SYSTEMIC** \| \| fatigue (n=20), headache (n=11), fever (n=5), chest pain (n=11), excessive sweating (n=1), chills (n=1) \| \|  \| \| **RESPIRATORY** \| \| dyspnea / breathlessness (n=20), cough (n=11), pulmonary fibrosis (n=3), lung hypoperfusion (n=1), impaired lung function (n=3), thromboembolism (n=4), sore throat (n=4), nasal congestion (n=2), sputum (n=3) \| \|  \| \| **CARDIOVASCULAR & HEMATOLOGICAL** \| \| palpitations & arrhythmias (n=7), peri-, myoperi- and myocarditis (n=2), tachycardia (n=2), cardiac stroke (n=1), venous/arterial thrombosis (n=1), myocardial inflammation (n=2), limb edema (n=2) \| \|  \| \| **NEUROLOGICAL & NEUROCOGNITIVE** \| \| hyperesthesia (n=1), loss or altered smell (n=13), loss or altered taste (n=13), numbness (n=1), muscle weakness (n=6), cognitive fatigue (n=1), apathy (n=1), stroke (n=2), neuropathy (n=2), myopathy (n=1), muscle pain (myalgia) (n=11), joint pain (arthralgia) (n=9), intracerebral hematoma (n=1), cerebral venous thrombosis (n=1), bladder incontinence (n=2), swallowing difficulties (n=1), encephalopathy (n=1), dizziness / vertigo (n=5), tinnitus (n=2), earache (n=1), visual disorders / eye redness (n=3), hearing loss (n=2), spasms (n=1), muscle atrophy (n=1), brain fog and memory loss (n=11), ), depression (n=6), sleep disorders (n=11), attention disorders (n=7), anxiety (n=7), posttraumatic symptoms (n=3), executive functioning difficulties (n=3), ataxia (n=1) \| \|  \| \|  \| \| **GASTROINESTINAL** \| \| general gastrointestinal complaints (n=4), diarrhea (n=6), vomiting (n=4), loss of appetite (n=5), nausea (n=5), abdominal pain (n=4), bowel incontinence (n=1), acid reflux (n=2), gastrointestinal bleeding (n=1), constipation (n=1) \| \|  \| \| **CUTANEOUS** \| \| skin rashes (n=6), alopecia (n=4) \| \|  \| |
| --- | --- | --- | --- | --- | --- | --- | --- | --- | --- | --- | --- | --- | --- | --- | --- | --- | --- | --- | --- | --- | --- |
