# Supplementary material for: Long COVID Through a Public Health Lens: An Umbrella Review
Source: Public Health Rev. 2022 Mar 15;43:1604501. doi: 10.3389/phrs.2022.1604501 (PMC8963488; doi:10.3389/phrs.2022.1604501)
Supplement: Supplementary file 2 [file DataSheet4.doc]

Supplementary file 4. Prevalence estimates reported in studies (follow-up ≥ 12 weeks) without control groups or population-based samples & their risk of bias assessment (Long COVID through a public health lens: An Umbrella Review. Switzerland 2021)

| Authors [Reference]  (# = preprint at time of data extraction) | Study Design | Cases (n=) | Hospitalized  (%) | Follow-up  (weeks) | Prevalence  (%) | Risk of Bias* |
| --- | --- | --- | --- | --- | --- | --- |
| Savarraj et al. ^1^ | Cohort | 58 | 100 | ≥12 | 71 | a. high risk |
|  |  |  |  |  |  | b. high risk |
|  |  |  |  |  |  | c. high risk |
| Venturelli et al. ^2^ | Cohort | 767 | 87 | ﻿12 (median) | 51.4 | a. high risk |
|  |  |  |  |  |  | b. high risk |
|  |  |  |  |  |  | c. high risk |
| Moreno-Perez et al.^3^ | Cohort | 277 | 66 | 10-14 | 50.9 | a. high risk |
|  |  |  |  |  |  | b. high risk |
|  |  |  |  |  |  | c. low risk |
| Sonnweber et al.^4^ | Cohort | 145 | 75 | > 14 | 41 | a. high risk |
|  |  |  |  |  |  | b. high risk |
|  |  |  |  |  |  | c. low risk |
| Buonsenso et al.^5^ | Survey | 129 | 7 | >17 | 51.5 | a. high risk |
|  |  |  |  |  |  | b. high risk |
|  |  |  |  |  |  | c. low risk |
| Arnold et al.^6^ | Cohort | 110 | 100 | 8-12 | 74 | a. high risk |
|  |  |  |  |  |  | b. high risk |
|  |  |  |  |  |  | c. low risk |
| Munblit et al.^7^ | Cohort | 2649 | 100 | 31 (median) | 47.1 | a. low risk |
|  |  |  |  |  |  | b. high risk |
|  |  |  |  |  |  | c. high risk |
| Davis et al.^8^ | Survey | 3762 | 8.4 | Up to 24 | 66.7 | a. high risk |
|  |  |  |  |  |  | b. high risk |
|  |  |  |  |  |  | c. high risk |
| Zhao et al.^9^ | Cohort | 55 | 100 | 12 | 64 | a. High risk |
|  |  |  |  |  |  | b. high risk |
|  |  |  |  |  |  | c. low risk |
| Lerum et al.^10^ | Cohort | 103 | 100 | 12 | 54 | a. high risk |
|  |  |  |  |  |  | b. high risk |
|  |  |  |  |  |  | c. low risk |
| ﻿Tabatabaei et al.^11^ | Cohort | 52 | 76.7 | 13 (mean) | 42.3 | a. high risk |
|  |  |  |  |  |  | b. high risk |
|  |  |  |  |  |  | c. low risk |
| ﻿Huang et al.^12^ | Cohort | 1733 | 100 | 26 (median) | 76 | a. high risk |
|  |  |  |  |  |  | b. high risk |
|  |  |  |  |  |  | c. low risk |
| Jacobson et al.^13^ | Cohort | 118 | 18.6 | 12-16 | 64.2 (non-hospitalized)  81.5 (hospitalized) | a. high risk |
|  |  |  |  |  |  | b. high risk |
|  |  |  |  |  |  | c. high risk |
| Perlis et al.^14^ # | Survey | 6211 | - | ≥ 24 | 2.2 | a. low risk |
|  |  |  |  |  |  | b. high risk |
|  |  |  |  |  |  | c. high risk |
| Han et al.^15^ | Cohort | 114 | 100 | 24 | 35 | a. high risk |
|  |  |  |  |  |  | b. high risk |
|  |  |  |  |  |  | c. low risk |
| Blanco et al.^16^ | Cohort | 100 | 100 | 15 (median) | 52 | a. high risk |
|  |  |  |  |  |  | b. high risk |
|  |  |  |  |  |  | c. high risk |
| Sykes et al.^17^ | Cohort | 134 | 100 | 16 (median) | 86 | a. high risk |
|  |  |  |  |  |  | b. high risk |
|  |  |  |  |  |  | c. high risk |
| Morin et al.^18^ | Cohort | 478 | 100 | 12- 16 | 51 | a. high risk |
|  |  |  |  |  |  | b. high risk |
|  |  |  |  |  |  | c. high risk |
| Horvath et al.^19^ | Cohort | 102 | 0 | 12 (mean) | 36 (smell alterations)  28 (taste alterations) | a. high risk |
|  |  |  |  |  |  | b. high risk |
|  |  |  |  |  |  | c. high risk |
| Bellan et al.^20^ | Cohort | 238 | 100 | 12-16 | 53.8 (functional impairment)  17.2 (PTSD symptoms) | a. high risk |
|  |  |  |  |  |  | b. high risk |
|  |  |  |  |  |  | c. high risk |
| Suárez-Robles et al.^21^ | Cohort | 134 | 100 | 13 | >40 | a. high risk |
|  |  |  |  |  |  | b. high risk |
|  |  |  |  |  |  | c. high risk |
| Simani et al.^22^ | Cohort | 120 | 100 | 24 | 17.5 (fatigue)  5.8 (PTSD) | a. high risk |
|  |  |  |  |  |  | b. high risk |
|  |  |  |  |  |  | c. low risk |
| Shah et al.^23^ | Cohort | 60 | 100 | 12 | 58 | a. high risk |
|  |  |  |  |  |  | b. high risk |
|  |  |  |  |  |  | c. high risk |
| Khalaf et al.^24^ # | Cohort | 538 | 51.3 | 12 | 84.6 | a. low risk |
|  |  |  |  |  |  | b. low risk |
|  |  |  |  |  |  | c. high risk |
| Townsend et al.^25^ | Cohort | 153 | 48 | 11 (median) | 62 | a. high risk |
|  |  |  |  |  |  | a. high risk |
|  |  |  |  |  |  | c. high risk |
| Darley et al.^26^ | Cohort | 78 | 12 | up to 16 | 39.7 | a. unclear |
|  |  |  |  |  |  | b. high risk |
|  |  |  |  |  |  | c. high risk |
| Wong et al.^27^ | Cohort | 78 | 100 | 12 | 76 | a. high risk |
|  |  |  |  |  |  | b. high risk |
|  |  |  |  |  |  | c. low risk |

*risk of bias assessment based on three items, adapted from Hoy et al (reference 15, manuscript).: a) is the target population representative of the national population; b) was some sort of random selection used to select the sample, OR was a census undertaken? c) was the likelihood on non-response bias minimal?

List of studies

1. Savarraj JP, et al. Pain and other neurological symptoms aare present at three-month after hospitalization in COVID-19 patients.

2. Venturelli S, Benatti S V., Casati M, et al. Surviving COVID-19 in Bergamo Province: A post-Acute outpatient re-evaluation.

3. Moreno-Perez O, Merino E, Leon-Ramirez J-M, et al. Post-acute COVID-19 syndrome. Incidence and risk factors: A Mediterranean cohort study.

4. Sonnweber T, Sahanic S, Pizzini A, et al. Cardiopulmonary recovery after COVID-19 – an observational prospective multi-center trial.

5. Buonsenso D, Munblit D, De Rose C, et al. Preliminary Evidence on Long COVID in children.

6. Arnold DT, Hamilton FW, Milne A, et al. Patient outcomes after hospitalisation with COVID-19 and implications for follow-up: Results from a prospective UK cohort.

7. Munblit D, Bobkova P, Spiridonova E, et al. Risk factors for long-term consequences of COVID-19 in hospitalised adults in Moscow using the ISARIC Global follow-up protocol: StopCOVID cohort study.

8. Davis HE, Assaf GS, McCorkell L, et al. Characterizing long COVID in an international cohort: 7 months of symptoms and their impact.

9. Zhao Y miao, Shang Y min, Song W bin, et al. Follow-up study of the pulmonary function and related physiological characteristics of COVID-19 survivors three months after recovery.

10. Lerum TV, Aaløkken TM, Brønstad E, et al. Dyspnoea, lung function and CT findings three months after hospital admission for COVID-19.

11. Tabatabaei SMH, Rajebi H, Moghaddas F, Ghasemiadl M, Talari H. Chest CT in COVID-19 pneumonia: what are the findings in mid-term follow-up?

12. Huang et al. 6-month consequences of COVID-19 in patients discharged from hospital: a cohort study.

13. Jacobson KB, et al. Patients with uncomplicated COVID-19 have long-term persistent symptoms and functional impairment similar to patients with severe COVID-19: a cautionary tale during a global pandemic.

14. Perlis RH, et al. Persistence of symptoms up to 10 months following acute COVID-19 illness.

15. Han X, Fan Y, Alwalid O, et al. Six-Month Follow-up Chest CT findings after Severe COVID-19 Pneumonia.

16. Blanco J, Navarro F, Sanjoaquin I, et al. Pulmonary long-term consequences of COVID-19 infections after hospital discharge.

17. Sykes DL, et al. Post-COVID-19 Symptom Burden: What is Long-COVID and How Should We Manage It?

18. Morin L, Savale L, Pham T, et al. Four-Month Clinical Status of a Cohort of Patients after Hospitalization for COVID-19.

19. Horvath L, Lim JWJ, Taylor JW, et al. Smell and taste loss in COVID-19 patients: assessment outcomes in a Victorian population.

20. Bellan M, Soddu D, Balbo PE, et al. Respiratory and psychophysical sequelae among patients with covid-19 four months after hospital discharge.

21. Suárez-Robles M, Iguaran-Bermúdez MDR, García-Klepizg JL, Lorenzo-Villalba N, Méndez-Bailón M. Ninety days post-hospitalization evaluation of residual covid-19 symptoms through a phone call check list.

22. Simani L, Ramezani M, Darazam IA, et al. Prevalence and correlates of chronic fatigue syndrome and post-traumatic stress disorder after the outbreak of the COVID-19.

23. Shah AS, Wong AW, Hague CJ, et al. A prospective study of 12-week respiratory outcomes in COVID-19-related hospitalisations.

24. Khalaf M, et al. Prevalence and predictors of persistent symptoms after clearance of SARS-CoV-2 infection: a report from Egypt.

25. Townsend L, Dowds J, O’Brien K, et al. Persistent Poor Health Post-COVID-19 Is Not Associated with Respiratory Complications or Initial Disease Severity.

26. Darley DR, Dore GJ, Cysique L, et al. Persistent symptoms up to four months after community and hospital-managed SARS-CoV-2 infection.

27. Wong AW, Shah AS, Johnston JC, Carlsten C, Ryerson CJ. Patient-reported outcome measures after COVID-19: A prospective cohort study.
