# Supplementary material for: Long COVID Through a Public Health Lens: An Umbrella Review
Source: Public Health Rev. 2022 Mar 15;43:1604501. doi: 10.3389/phrs.2022.1604501 (PMC8963488; doi:10.3389/phrs.2022.1604501)
Supplement: Supplementary file 3 [file DataSheet5.docx]

Supplementary file 5. Risk of bias assessment of studies (follow-up ≥ 12 weeks) reporting prevalence estimates and including control groups and/or population-based samples (Long COVID through a public health lens: An Umbrella Review. Switzerland 2021)

| Authors [Reference, as in manuscript]* | Risk of Bias |
| --- | --- |
| Cirulli et al. (1) | a. high risk |
|  | b. high risk |
|  | c. high risk |
| Desgranges et al. (2) | a. high risk |
|  | b. high risk |
|  | c. low risk |
| Graham et al. (3) | a. high risk |
|  | b. high risk |
|  | c. low risk |
| Havervall et al. (4) | a. high risk |
|  | b. high risk |
|  | c. high risk |
| Logue et al. (5) | a. high risk |
|  | b. high risk |
|  | c. low risk |
| Menges et al. (6) | a. low risk |
|  | b. low risk |
|  | c. high risk |
| Miller et al. (7) | a. high risk |
|  | b. high risk |
|  | c. high risk |
| Molteni et al. (8) | a. high risk |
|  | b. high risk |
|  | c. high risk |
| Petersen et al. (9) | a. low risk |
|  | b. low risk |
|  | c. low risk |
| Radtke et al. (10) | a. low risk |
|  | b. low risk |
|  | c. high risk |
| Stavem et al. (11) | a. high risk |
|  | b. high risk |
|  | c. high risk |
| Sudre et al. (12) | a. high risk |
|  | b. high risk |
|  | c. high risk |
| Xiong et al. (13) | a. high risk |
|  | b. high risk |
|  | c. low risk |

*risk of bias assessment based on three items, adapted from Hoy et al (reference 15, manuscript).: a) is the target population representative of the national population; b) was some sort of random selection used to select the sample, OR was a census undertaken? c) was the likelihood on non-response bias minimal?

1. Cirulli ET, et al. Long-term COVID-19 symptoms in a large unselected population. medRxiv. 2020.

2. Desgranges F, Tadini E, Munting A, Regina J, Filippidis P, Viala B, et al. Post ‑ COVID ‑ 19 syndrome in outpatients : a cohort study. medRxiv. 2021.

3. Graham EL, Clark JR, Orban ZS, Lim PH, Szymanski AL, Taylor C, et al. Persistent neurologic symptoms and cognitive dysfunction in non-hospitalized Covid-19 “long haulers.” Ann Clin Transl Neurol. 2021;8(5):1073–85.

4. Havervall S, Rosell A, Phillipson M, Mangsbo SM, Nilsson P, Hober S, et al. Symptoms and Functional Impairment Assessed 8 Months After Mild COVID-19 Among Health Care Workers. JAMA [Internet]. 2021;11. Available from: http://www.ncbi.nlm.nih.gov/pubmed/33825846

5. Logue JK, Franko NM, McCulloch DJ, McDonald D, Magedson A, Wolf CR, et al. Sequelae in Adults at 6 Months After COVID-19 Infection. JAMA Netw open. 2021;4(2):e210830.

6. Menges D, Ballouz T, Anagnostopoulos A, Aschmann HE, Domenghino A, Fehr JS, et al. Burden of post-COVID-19 syndrome and implications for healthcare service planning: A population-based cohort study. PLoS One. 2021;16(7 July):1–19.

7. Miller F, Nguyen V, Navaratnam AMD, Shrotri M, Kovar J, Hayward AC, et al. Prevalence of persistent symptoms in children during the COVID-19 pandemic: evidence from a household cohort study in England and Wales. medRxiv [Internet]. 2021;2021.05.28.21257602. Available from: http://medrxiv.org/content/early/2021/06/02/2021.05.28.21257602.abstract

8. Molteni E, Sudre CH, Canas LS, Bhopal SS. Illness duration and symptom profile in a large cohort of symptomatic UK school-aged children tested for SARS- CoV-2. medRxiv. 2021;

9. Petersen MS, et al. Long COVID in the Faroe Islands - a longitudinal study among non-hospitalized patients. Clin Inf. 2020;

10. Radtke T, Ulyte A, Puhan MA, Kriemler S. Long-term SymptomsAfter SARS-CoV-2 Infection in Children and Adolescents. JAMA. 2021;326(9):2603–15.

11. Stavem K, Ghanima W, Olsen MK, Gilboe HM, Einvik G. Persistent symptoms 1.5-6 months after COVID-19 in non-hospitalised subjects: A population-based cohort study. Thorax. 2021;76(4):405–7.

12. Sudre CH, Murray B, Varsavsky T, Graham MS, Penfold RS, Bowyer RC, et al. Attributes and predictors of long COVID. Nat Med. 2021;27(4):626–31.

13. Xiong Q, Xu M, Li J, Liu Y, Zhang J, Xu Y, et al. Clinical sequelae of COVID-19 survivors in Wuhan, China: a single-centre longitudinal study. Clin Microbiol Infect. 2021;27(1):89–95.
