# Supplementary material for: Long COVID Through a Public Health Lens: An Umbrella Review
Source: Public Health Rev. 2022 Mar 15;43:1604501. doi: 10.3389/phrs.2022.1604501 (PMC8963488; doi:10.3389/phrs.2022.1604501)
Supplement: Supplementary file 4 [file DataSheet2.doc]

Supplementary file 2. Assessing the methodological quality of systematic review (AMSTAR) Scores – Reviews (Long COVID through a public health lens: An Umbrella Review. Switzerland 2021)

| Title and Reference | AMSTER Score |
| --- | --- |
| Case report and systematic review suggest that children may experience similar long-term effects to adults after clinical COVID-19 (1) | Critically low quality |
| COVID-19 sequelae in adults aged less than 50 years: A systematic review (2) | Moderate quality |
| Rehabilitation and COVID-19: a rapid living systematic review by Cochrane Rehabilitation Field updated as of December 31st, 2020 and synthesis of the scientific literature of 2020 (3) | Moderate quality |
| Proposed delay for safe surgery after COVID-19 (4) | Moderate quality |
| Late Complications of COVID-19; a Systematic Review of Current Evidence (5) | Low quality |
| Characterising long-term covid-19: a rapid living systematic review (6) | Moderate quality |
| The ocurrence of long COVID: a rapid review (7) | Critically low |
| Long COVID, a comprehensive systematic scoping review (8) | Critically low |
| Living with COVID19. Second Review (9) | Critically low |
| Epidemiology of Long Covid. A Pragmatic Review of the Literature (10) | Critically low |
| Post-COVID-19 Syndrome: The Persistent Symptoms at the Post-viral Stage of the Disease. A Systematic Review of the Current Data (11) | Moderate |
| Post-acute COVID-19 syndrome (12) | Critically low |
| Long-COVID and Post-COVID Health Complications: An Up-to-Date Review on Clinical Conditions and Their Possible Molecular Mechanisms (13) | Critically low |
| Characteristics and predictors of acute and chronic post-COVID syndrome: A systematic review and meta-analysis (14) | Moderate |
| Long COVID and Myalgic Encephalomyelitis/Chronic Fatigue Syndrome (ME/CFS)—A Systemic Review and Comparison of Clinical Presentation and Symptomatology (15) | Critically low |
| Frequency, signs and symptoms, and criteria adopted for long COVID: a systematic review (16) | Moderate |
| Long COVID or post-COVID-19 syndrome: putative pathophysiology, risk factors, and treatments (17) | Low quality |
| Assessment of the Frequency and Variety of Persistent Symptoms Among Patients With COVID-19 (18) | Moderate quality |
| Cardio-Pulmonary Sequelae in Recovered COVID-19 Patients: Considerations for Primary Care (19) | Low quality |
| Global prevalence of prolonged gastrointestinal symptoms in  COVID-19 survivors and potential pathogenesis: A systematic  review and meta-analysis (20) | Low quality |
| Prevalence of post-COVID-19 symptoms in hospitalized and non-hospitalized COVID-19 survivors: A systematic review and meta-analysis (21) | High quality |
| More than 50 Long-term effects of COVID-19: a systematic review and meta-analysis (22) | Moderate quality |

Based on: Shea BJ, Reeves BC, Wells G, Thuku M, Hamel C, Moran J, Moher D, Tugwell P, Welch V, Kristjansson E, Henry DA. AMSTAR 2: a critical appraisal tool for systematic reviews that include randomised or non-randomised studies of healthcare interventions, or both. BMJ. 2017 Sep 21;358:j4008.

List of studies:

1. Ludvigsson JF. Case report and systematic review suggest that children may experience similar long-term effects to adults after clinical COVID-19.

2. Willi S, Lüthold R, Hunt A, Hänggi NV, Sejdiu D, Scaff C, et al. COVID-19 sequelae in adults aged less than 50 years: A systematic review.

3. Medica EM, Sire ADE, Andrenelli E, Negrini F, Patrini M, Lazzarini SG, et al. Rehabilitation and COVID-19 : a rapid living systematic review by Cochrane Rehabilitation Field updated as of December 31st , 2020 and synthesis of the scientific literature.

4. Kovoor JG, Scott NA, Tivey DR, Babidge WJ, Scott DA, Beavis VS, et al. Proposed delay for safe surgery after COVID-19.

5. SeyedAlinaghi S, Afsahi AM, MohsseniPour M, Behnezhad F, Salehi MA, Barzegary A, et al. Late Complications of COVID-19; a Systematic Review of Current Evidence.

6. Michelen M, Manoharan L, Elkheir N, Cheng V, Dagens A, Hastie C, et al. Characterising long COVID: A living systematic review.

7. Iwu CJ, Iwu CD, Wiysonge CS. The occurrence of long COVID: a rapid review.

8. Akbarialiabad H, Taghrir MH, Abdollahi A, Ghahramani N, Kumar M, Paydar S, et al. Long COVID, a comprehensive systematic scoping review.

9. NIHR. Living with COVID19. Second Review [Internet].

10. Zapatero DC, Hanquet G, Heede K Van Den. Epidemiology of Long Covid : a Pragmatic Review of the Literature.

11. Salamanna F, Veronesi F, Martini L, Landini MP, Fini M. Post-COVID-19 Syndrome: The Persistent Symptoms at the Post-viral Stage of the Disease. A Systematic Review of the Current Data.

12. Nalbandian A, Sehgal K, Gupta A, Madhavan M V., McGroder C, Stevens JS, et al. Post-acute COVID-19 syndrome.

13. Andrade BS, Siqueira S, de Assis Soares WR, de Souza Rangel F, Santos NO, Dos Santos Freitas A, et al. Long-covid and post-covid health complications: An up-to-date review on clinical conditions and their possible molecular mechanisms.

14. Iqbal FM, Lam K, Sounderajah V, Clarke JM, Ashrafian H, Darzi A. Characteristics and predictors of acute and chronic post-COVID syndrome: A systematic review and meta-analysis.

15. Wong TL, Weitzer DJ. Long COVID and myalgic encephalomyelitis/chronic fatigue syndrome (ME/CFS)-A systemic review and comparison of clinical presentation and symptomatology.

16. Cabrera Martimbianco AL, Pacheco RL, Bagattini ÂM, Riera R. Frequency, signs and symptoms, and criteria adopted for long COVID: a systematic review.

17. Yong SJ. Long COVID or post-COVID-19 syndrome: putative pathophysiology, risk factors, and treatments.

18. Nasserie T, Hittle M, Goodman SN. Assessment of the Frequency and Variety of Persistent Symptoms among Patients with COVID-19: A Systematic Review.

19. Sarfraz Z, Sarfraz A, Barrios A, Garimella R, Dominari A, KC M, et al. Cardio-Pulmonary Sequelae in Recovered COVID-19 Patients: Considerations for Primary Care.

20. Yusuf F, Fahriani M, Mamada SS, Frediansyah A, Abubakar A, Maghfirah D, et al. Global prevalence of prolonged gastrointestinal symptoms in COVID-19 survivors and potential pathogenesis: A systematic review and meta-analysis.

21. Fernández-de-las-Peñas C, Palacios-Ceña D, Gómez-Mayordomo V, Florencio LL, Cuadrado ML, Plaza-Manzano G, et al. Prevalence of post-COVID-19 symptoms in hospitalized and non-hospitalized COVID-19 survivors: A systematic review and meta-analysis.

22. Lopez-Leon et al. More More than 50 Long-term effects of COVID-19: a systematic review and meta-analysis.
