# Supplementary material for: Long COVID Through a Public Health Lens: An Umbrella Review
Source: Public Health Rev. 2022 Mar 15;43:1604501. doi: 10.3389/phrs.2022.1604501 (PMC8963488; doi:10.3389/phrs.2022.1604501)
Supplement: Supplementary file 5 [file DataSheet1.docx]

Supplementary file 1. Review Search Strategy (Long COVID through a public health lens: An Umbrella Review. Switzerland 2021)

| Strategy |
| --- |
| MEDLINE + CINAHL (Ebscohost)  (COVID-19 OR covid OR SARS-CoV-2. ab) AND (symptom* OR "clinical feature*" OR characteristic* OR sequela* OR complication* OR impact OR implication* OR consequence* OR effect*.ab) AND ("long-term symptom*" OR "long-term clinical features" OR "long-term signs" OR "long-term characteristic*" OR "long-term sequela*" OR "long-term complication*" OR "long-term impact" OR "long-term implication*" OR "long-term consequence*" OR "long-term effect*" OR post-acute OR long-tail OR chronic-COVID OR long-COVID or "long COVID" OR "long-term COVID" OR post-COVID OR recurrent OR lingering OR persist* OR post-discharge Or "post discharge" OR "prolonged symptom*" OR post-chronic OR long-haul* .ab) AND ("systematic review" OR "literature review" OR "review" OR "meta-analysis" .ab) from 2020 on |
| WHO COVID (incl. Elsevier, medRxiv)  (tw:(COVID-19 OR covid OR SARS-CoV-2)) AND (tw:(symptom* OR "clinical feature*" OR characteristic* OR sequela* OR complication* OR impact OR implication* OR consequence* OR effect*)) AND (tw:("long-term symptom*" OR "long-term clinical features" OR "long-term signs" OR "long-term characteristic*" OR "long-term sequela*" OR "long-term complication*" OR "long-term impact" OR "long-term implication*" OR "long-term consequence*" OR "long-term effect*" OR post-acute OR long-tail OR chronic-COVID OR long-COVID or "long COVID" OR "long-term COVID" OR post-COVID OR post-discharge Or "post discharge" OR "prolonged symptom*" OR post-chronic OR long-haul*)) AND (tw:("systematic review" OR "literature review" OR "review" OR "meta-analysis")) AND c( WHO COVID, ELSEVIER, medRxiv (exclude Medline) |
| Embase  ('covid-19':ab,ti OR 'covid':ab,ti OR 'sars-cov2':ab,ti OR 'chronic-covid':ab,ti OR 'long-covid':ab,ti OR 'long covid':ab,ti OR 'long-term covid':ab,ti OR 'post-covid':ab,ti) AND ('symptom*':ab,ti OR 'clinical feature*':ab,ti OR 'characteristic*':ab,ti OR 'sequela*':ab,ti OR 'complication*':ab,ti OR 'impact':ab,ti OR 'implication*':ab,ti OR 'consequence*':ab,ti OR 'effect*':ab,ti) AND ('long-term symptom*':ab,ti OR 'long-term clinical features':ab,ti OR 'long-term signs':ab,ti OR 'long-term characteristic*':ab,ti OR 'long-term sequela*':ab,ti OR 'long-term complication*':ab,ti OR 'long-term impact':ab,ti OR 'long-term implication*':ab,ti OR 'long-term consequence*':ab,ti OR 'long-term effect*':ab,ti OR 'post-acute':ab,ti OR 'long-tail':ab,ti OR 'prolonged symptom*':ab,ti OR 'post-chronic':ab,ti OR 'long-haul*':ab,ti) AND ('systematic review':ab,ti OR 'literature review':ab,ti OR 'review':ab,ti OR 'meta-analysis':ab,ti) (exclude Medline) |
